# The effect of an abdominal binder on postoperative outcome after open incisional hernia repair in sublay technique: a multicenter, randomized pilot trial (ABIHR-II)

**DOI:** 10.1007/s10029-023-02838-4

**Published:** 2023-07-19

**Authors:** P. R. Ortiz, E. Lorenz, F. Meyer, R. Croner, S. Lünse, R. Hunger, R. Mantke, A. Benz-Weisser, K. Zarras, M. Huenerbein, C. Paasch

**Affiliations:** 1 Charité Universitätsmedizin-Berlin, Berlin, Germany; 2 Department of General, Abdominal and Cancer Surgery, Helios Klinikum Berlin-Buch, Berlin, Germany; 3 Department of General, Abdominal, Vascular and Transplant Surgery, Otto-Von-Guericke University Hospital Magdeburg, Magdeburg, Germany; 4 Department of General and Abdominal Surgery, Clinic for General and Abdominal Surgery, University Hospital Brandenburg an der Havel, Hochstrasse 29, 14770 Brandenburg an der Havel, Germany; 5 Faculty of Medicine, Brandenburg Medical School Theodor Fontane, Brandenburg, Germany; 6 Faculty of Health Sciences Brandenburg, Brandenburg Medical School Theodor Fontane, Brandenburg an der Havel, Germany; 7 Department of General, Abdominal Vascular and Transplant Surgery, Westpfalz-Klinikum GmbH, Kaiserslautern, Germany; 8 Department of Abdominal Minimally Invasive and Cancer Surgery, Marien Hospital Düsseldorf, Düsseldorf, Germany; 9 Department of Surgery, Oberhavel Clinic Oranienburg, Oranienburg, Germany

**Keywords:** Abdominal binder, Incisional hernia, Open sublay repair, Hernia

## Abstract

**Introduction:**

Although the evidence is minimal, an abdominal binder is commonly prescribed after open incisional hernia repair (IHR) to reduce pain. This study aimed to investigate this common postoperative treatment.

**Methods:**

The ABIHR-II trial was a national prospective, randomized, multicenter non-AMG/MPG pilot study with two groups of patients (wearing an abdominal binder (AB) for 2 weeks during daytime vs. not wearing an AB following open IHR with the sublay technique). Patient enrollment took place from July 2020 to February 2022. The primary endpoint was pain at rest on the 14th postoperative day (POD) using the visual analog scale (VAS). The use of analgesics was not systematically recorded. Mixed-effects linear regression models were used.

**Results:**

A total of 51 individuals were recruited (25 women, 26 men; mean age 61.4 years; mean body mass index 30.65 kg/m^2^). The per-protocol analysis included 40 cases (AB group, *n* = 21; No-AB group, *n* = 19). Neither group showed a significant difference in terms of pain at rest, limited mobility, general well-being, and seroma formation and rate. Patients among the AB group had a significantly lower rate of surgical site infection (SSI) on the 14th POD (AB group 4.8% (*n* = 1) vs. No-AB group 27.8% (*n* = 5), *p* = 0.004).

**Conclusion:**

Wearing an AB did not have an impact on pain and seroma formation rate but it may reduce the rate of postoperative SSI within the first 14 days after surgery. Further trials are mandatory to confirm these findings.

**Supplementary Information:**

The online version contains supplementary material available at 10.1007/s10029-023-02838-4.

## Introduction

Incisional hernias frequently occur after midline incisions with a prevalence of up to 35.6% [1]. Their treatment, therefore, has a significant socioeconomic impact. Individuals who have undergone open or minimally invasive incisional hernia repair (IHR) often experience postoperative pain, seroma formation, and immobility. Surgeons often recommend wearing an abdominal binder (AB) with the belief that it may improve the outcomes of IHR. [2–4]. Thus, in a survey of 44 surgical departments in Germany, our study group found that a majority of 31 departments prescribed an AB after IHR [2]. But the evidence in the literature is low. Rothmann et al. (2014) published a systematic review on AB-use following laparotomies (8 studies, *n* = 578) [4]. The authors concluded that the effects on postoperative pain after laparotomy and seroma formation after ventral hernia repair remain unclear. A prospective randomized clinical trial was considered mandatory.

To further investigate the impact of an AB, a study was conducted from 2017 to 2018 with 163 individuals who underwent IHR. Overall, 71.2% of patients reported that AB reduced pain after surgery. A prolonged period of wearing an AB had no statistical significance on postoperative morbidity [3]. The findings of these surveys led to the conduction of the ABIHR-I trial (*n* = 40), a multicenter randomized pilot project. The primary endpoint was pain after laparoscopic IHR with the intraperitoneal onlay mesh technique on the 14th POD. The patients in the AB group had significantly less postoperative pain [5]. The effect of wearing an AB on the rate of postoperative complications such as pain and seroma formation after sublay IHR has not been investigated in a randomized clinical trial. This surgical approach is widely used. Hence, improvement of postoperative treatment is mandatory.

For this reason, this study was conducted. The study was designed along the lines of the ABIHR-I study to allow a meaningful comparison.

## Methods

The ABIHR-II trial was a national prospective, randomized, multicenter non-AMG/MPG pilot study with two groups of patients (wearing an abdominal binder (AB) for 2 weeks during daytime vs. not wearing an AB following open IHR with the sublay technique. Patient enrollment took place from July 2020 to February 2022.

The randomized clinical trial was conducted at the following hospitals: HELIOS Hospital Berlin-Buch (Germany), Otto-von-Guericke University Hospital Magdeburg (Germany), University Hospital Brandenburg an der Havel (Germany), Marien Hospital Düsseldorf (Germany), and Westpfalz Hospital Kaiserslautern (Germany).

The study received primary approval from the Ethics Committee of the Berlin Medical Association on July 11, 2020 (Eth-09/20) and was conducted in accordance with the ethical standards of the Declaration of Helsinki 1975.

An ethics vote was successfully obtained from all responsible German state medical associations (2022, Brandenburg; 2021, Rhineland-Palatinate; 2021, North Rhine Westphalia; 2020 Saxony Anhalt).

The study was registered with the German Clinical Trials Registry (DRKS00017410) and funded by the 2020 Research Grant from the European Hernia Society.

**Fig. 1:**
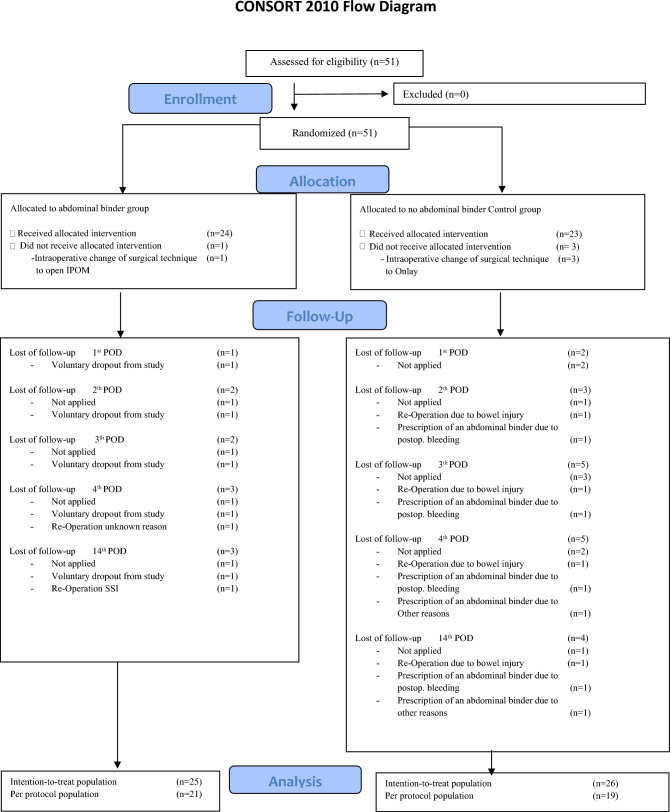
The CONSORT 2010 flow chart of the ABIHR-II trial is depicted

The ABIHR-II trial was conducted according to the CONSORT 2010 statement (Fig. 1) [6].

### Study population

#### Inclusion criteria

Patients suffering from incisional hernia and scheduled for open elective sublay repair with hernia gap closure were enrolled in the study. A minimum age of 18 years at the time of surgery was required for participation in the study. An upper age limit was not specified.

#### Exclusion criteria

Patients who were pregnant or had HIV infection were excluded from the study. In cases where the gap was not closed, individuals were counted as dropouts.

#### Primary endpoint

Pain at rest two weeks postoperatively was chosen as the primary endpoint of the study. The VAS score was used to measure pain.

#### Secondary endpoints

SSI (clinical examination with detection of redness, warming, swelling [7]), seroma formation (ultrasound imaging), the early recurrence rate (clinical examination), well-being (VAS scoring), and mobility (VAS scoring) on the 1st, 2nd, 3rd, 4th, and 14th POD were secondary endpoints. Further objectives were pain at rest (VAS scoring) on the 1st, 2nd, 3rd, 4th, and 14th POD, the length of hospital stays and reoperation rate. Mesh size, operating time, and surgeons' experience were also analyzed as we understand this could be an important variable for the results.

The secondary endpoint, recurrence rate 12 months after surgery as part of the study protocol, will be determined and published in the future.

#### Surgical approach

Sublay repair was performed according to the approach described by Rives and Stoppa [8]. A non-absorbable mesh was placed on the posterior wall of the rectus sheath. The choice of suture material was made individually by the surgeons in each hospital. This common open approach was chosen for the study because until this point we had only investigated AB prescriptions after laparoscopic repair [5].

Hernia gap closure was attempted in all included subjects, as there is evidence in the literature that a closure leads to less adverse hernia-site events [9]. In cases in which an intraoperative decision was made to repair the incisional hernia with another technique or in which hernia gap closure was not performed, the patient was considered a dropout.

#### Analgesic medication

The ABIHR-II analgesic regimen during patients' hospital stay consisted of oral ibuprofen 600 mg (1-1-1) or metamizole 500 mg (2-2-2), oxycodone 10 mg (1-0-1), and as-needed medication with piritramide subcutaneously. No actual consumption of pain medications was noted.

#### Randomization

Randomization lists were used and pseudorandom numbers were generated in R (ver. 4.0.2). The principal investigator performed randomization (1:1) after patients gave informed consent. No stratification took place.

#### Statistical analysis

Individuals who were randomized were known as the intention-to-treat population. Patients with data for the primary endpoint of pain at rest on the 14th POD were known as the per-protocol population. Baseline comparison in age, sex, BMI, operating time, and ASA class between study arms was performed using the chi-square test (with Yates’ continuity correction) and independent t-Test for categorical and continuous variables, respectively. The strength of effects was assessed by Cohen’s d and Cramer’s V.

To incorporate the longitudinal design of repeated measurements, a mixed-model analysis was performed in R (version 4.2.3, R Software Foundation) using the lme4-package [10].

The main effects of time and study, as well as an interaction between time and study group were examined. The patient was entered as a random factor in the model. Partial eta-squared (ηp2) was calculated as effect size, indicating the proportion of variance in the dependent variable that can be explained by the independent variable, with values around 0.01, 0.06, 0.14 interpreted as small, medium, and large effects, respectively [11]. A *p* value < 0.05 indicated statistical significance.

## Results

A total of 51 individuals were enrolled (25 females, 26 males). The mean age was 61.4 years and the body mass index was 31 kg/m^2^. The majority of patients had an American Society of Anesthesiologists (ASA) score of II. (ASA score I, *n* = 1; ASA score II, *n* = 33; ASA score III, *n* = 17). Ten patients suffered from a first relapse of an incisional hernia. The median operating time was 140 min (± 59). The duration of hospital stay was on average 8.7 days (± 7.6; table S1). A total of 8 individuals received a component separation and in 5 cases, a peridural catheter was placed (No-AB group, *n* = 3; AB group, *n* = 1; per-protocol population, *n* = 4). No early relapse occurred within the 14 PODs. In 5 cases, a reoperation took place (bowl injury, *n* = 1; hematoma, *n* = 2; SSI, *n* = 1; anastomotic leak, *n* = 1). No radiological intervention was documented. A total of 22 out of 45 patients were diagnosed with seroma formation (missing data: 6; per-protocol population: AB group *n* = 13, No-AB group *n* = 9).

The classification of incisional hernias by the European Hernia Society is depicted in Table S2. Among the per-protocol population (*n* = 21), a total of 8 patients suffered from a W1 hernia (hernia width < 4 cm; AB group: *n* = 4 vs. No-AB group *n* = 4), 21 from a W2 (hernia width ≥ 4–10 cm; AB group: *n* = 11 vs. No-AB group *n* = 10), and 11 from a W3 hernia (hernia width ≥ 10 cm; AB group: *n* = 6 vs. No-AB group *n* = 5).

### Univariate analysis of baseline characteristics and perioperative data

The univariate analysis of baseline characteristics of the intention-to-treat population is shown in table S1. The two groups did not differ significantly in any variable.

The univariate analysis of baseline characteristics of the per-protocol population is depicted in Table 1. The two groups did not differ significantly in any variable.

**Table 1:** Univariate analysis of patient characteristics and perioperative data (Per-protocol population)

Variable	*n* = 40^1^	No-AB group, *n* = 19^1^	AB group, *n* = 21^1^	*p* value^2^	t/X^2^ (df)	Effect size
Gender				0.97	0.12 (1)	0.055
Female	18 (45.0%)	8 (42.1%)	10 (47.6%)			
Male	22 (55.0%)	11 (57.9%)	11 (52.4%)			
Age years	63.4 (13.7)	62.4 (13.8)	64.2 (13.9)	0.67	− 0.43 (38)	− 0.135
BMI kg/m^2^	30.4 (5.4)	30.6 (6.0)	30.2 (4.9)	0.80	0.26 (38)	0.082
ASA score				0.25	2.74 (2)	0.262
I	1 (2.50%)	1 (5.26%)	0 (0%)			
II	24 (60.0%)	13 (68.4%)	11 (52.4%)			
III	15 (37.5%)	5 (26.3%)	10 (47.6%)			
Operating time minutes	138.1 (58.7)	133.7 (47.6)	142.1 (68.1)	0.65	− 0.45 (38)	− 0.144
Duration of hospital stay days	7.7 (4.5)	7.9 (3.5)	7.6 (5.3)	0.82	0.23 (38)	0.072
Surgeons experience years				0.50	4.38 (5)	0.331
< 5	1 (2.50%)	0 (0%)	1 (4.76%)			
5–10	10 (25.0%)	4 (21.1%)	6 (28.6%)			
> 10	9 (22.5%)	6 (31.6%)	3 (14.3%)			
> 20	15 (37.5%)	7 (36.8%)	8 (38.1%)			
> 30	1 (2.5%)	1 (5.26%)	0 (0%)			
> 40	4 (10.%)	1 (5.26%)	3 (14.3%)			
Mesh size cm^2^	569.0 (327.9)	675.7 (354.4)	472.4 (275.6)	0.052	2.04 (38)	0.641
Component separation	7 (17.9%)	4 (21.1%)	3 (15.0%)	0.94	0.24 (1)	0.079
Missing	1	0	1			
Relapse if an incisional hernia				0.13	7.18 (4)	0.424
No	34 (85.0%)	17 (89.5%)	17 (81.0%)			
Yes	6 (15.0%)	2 (10.5%)	4 (19.0%)			
Reoperation rate	3 (7.6%)	1 (5.3%)	2 (10.0%)			
Missing	1	0	1	> 0.99	0.31 (1)	0.089

^1^*n* (%); mean value (standard deviation)

^2^Chi-Quadrat Test for independence; *T* test

*AB* abdominal binder, *ASA* American society of anesthesiologists, *BMI* body mass index

Data are presented as mean (standard deviation) for continuous variables or total number (percentages) for categorical variables

Effect sizes are presented as Cohen’s d and Cramer’s V for independent t-tests and contingency tables, respectively

### Mixed-model analysis of endpoints among the per-protocol population

**Fig. 2:**
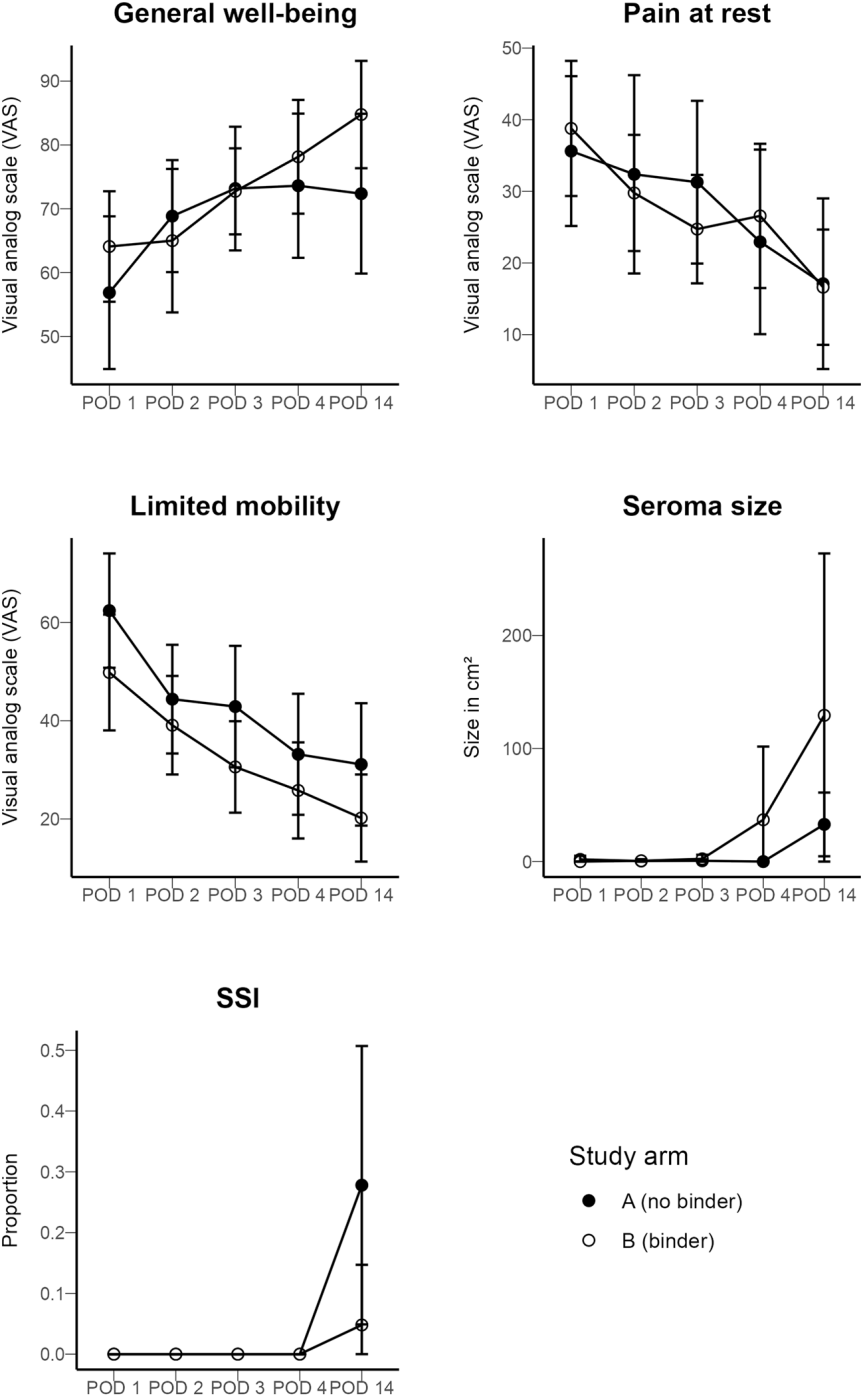
General well-being, limited mobility, pain at rest, and the seroma size on the 1st, 2nd, 3rd, 4th, and 14th POD of the per-protocol population (*n* = 21) is depicted. General well-being, limited mobility, and pain at rest were measured using a visual analog scale (VAS, y-axis). The seroma size (y-axis, cm^3^) was documented using ultrasound imaging. The appearance of an SSI was documented as *Yes* or *No*

Table S3 provides detailed information on endpoint data of the per-protocol population. Neither group differed with significance in terms of pain at rest on the 14th POD (AB group, 17.1; No-AB group, 16.6). Individuals in the AB group had a higher rate and size of seroma formation on the 14th POD (AB group, *n* = 13 (66.6%); No-AB group, *n* = 9 (50%), Fig. 2). For every endpoint, a highly significant time effect was observed with intermediate to strong effect sizes. For pain at rest, limited mobility, and general well-being, this indicates the general recovery after surgery. For seroma size and SSI, the time effect represents the time needed for complication development. The group effect was not significant for any of the endpoints, albeit falls short of significance for SSI with *p* = 0.05007. However, except for pain at rest, effect size metrics indicate a medium group effect. A significant interaction between group and time for SSI was observed, with a substantially higher rate of SSI in the No-AB group (AB group 4.8%, (*n* = 1) vs. No-AB group 27.8% (*n* = 5), *p* = 0.004, Table 2). Neither significant nor substantial interactions were observed for the other endpoints.

**Table 2:** Mixed-model analysis of endpoints among the per-protocol population

Endpoint	Interaction group × time			Main effect of group			Main effect of time		
	F (df_n_, df_d_)	*p*	η_p_^2^	F (df_n_, df_d_)	*p*	η_p_^2^	F (df_n_, df_d_)	*p*	η_p_^2^
Pain at rest	0.39 (4, 145)	0.814	0.011	0.01 (1, 38.1)	0.911	0	9.72 (4, 145)	< 0.001	0.211
Limited mobility	0.4 (4, 145)	0.810	0.011	3.1 (1, 38.3)	0.086	0.075	21.9 (4, 145)	< 0.001	0.377
General well-being	0.85 (4, 145)	0.496	0.023	1.44 (1, 37.8)	0.238	0.037	8.91 (4, 145)	< 0.001	0.197
Seroma size	1.25 (4, 148.3)	0.291	0.033	2.52 (1, 39.3)	0.120	0.06	3.34 (4, 148.3)	0.012	0.083
SSI	4.08 (4, 156.1)	0.004	0.095	4.03 (1, 49.8)	0.050	0.075	8.15 (4, 156.1)	< 0.001	0.173

*SSI* surgical site infection

## Discussion

To our knowledge, the present ABIHR-II project is the first study to investigate the effects of AB on SSI rate, seroma formation, limited mobility, general well-being, and pain at rest after open IHR with the sublay technique. AB is frequently prescribed and the open sublay procedure is frequently performed. Therefore, the analysis of these endpoints seems to be important to further optimize the postoperative course [2, 12].

The SSI rate after IHR with the sublay technique is about 15–20% [13, 14]. In this study, a significant interaction between group and time for SSI was observed, with a substantially higher rate of SSI in the No-AB group (AB group 4.8%, (*n* = 1) vs. No-AB group 27.8% (*n* = 5), *p* = 0.004, Table 2). It can be assumed that AB leads to less movement within the wound and stress. This could facilitate more sufficient wound healing. Translational studies with the determination of inflammatory markers from the surgical site could lead to an explanation. With this in mind, the prescription to wear an AB day and night can lead to a further reduction of SSI after IHR with the sublay technique. On the other hand, SSIs often occur within 30 days after surgery. We only collected data until the 14th POD. Therefore, the SSI rate might be higher and our findings should not be over-interpreted [7].

Seroma formation is common after open IHR. According to an analysis of the Herniamed registry, the seroma formation rate in 3965 patients who underwent sublay IHR is approximately 5% [12]. Even much higher rates are reported in the literature [15]. We found a seroma formation rate of 43.3% among the intention-to-treat population (55% among per-protocol population). But these findings are not plausible. Even small fluid formations on the 14th POD (1cm^3^) were considered to be a seroma formation in the study at hand. One could argue that measurement of a seroma formation should take place beyond one month after surgery. In line with clinical experience, Kaafarani et al. (2009) chose the rate of seroma formation 8 weeks after IHR as the primary endpoint of a randomized clinical trial [15].

It is conceivable that AB helps to prevent seroma formation, as it can reduce the size of the hernia sac and the future seroma space [16]. Little is known about the impact of AB on these surgical site occurrences [4]. A few previous trials including the ABIHR-I trial did not reveal any effect of AB on seroma formation [5, 16]. Patients in the AB group and the No-AB group of the present ABIHR-II study did not differ significantly in terms of seroma size (Table 2) and rate. Although again expected, seroma size was greater in the AB group without reaching significant value. In summary, it seems likely that AB plays no role in reducing seroma formation after IHR.

It can be postulated that AB may limit mobility after surgery. Even muscle atrophy due to lack of abdominal muscle use from wearing an AB has been discussed [17]. Hence, a survey among patients who underwent IHR revealed that 32.6% stated AB-induced mobility [3]. In this study, no statistically significant difference was revealed between both study groups in terms of limited mobility. But there was a tendency towards better mobility among patients in the AB group. The same observation without significance was made in the ABIHR-I study when only patients undergoing IHR with intraperitoneal mesh were analyzed [5]. These observations are supported by the results of a meta-analysis from China by Jiang et al. (2021). The authors reviewed 10 randomized clinical trials including 968 individuals who had undergone laparotomy, mostly cesarean incisions. They found significantly better performance on the 6-min walk test when wearing an AB [18]. Better mobility may lead to higher general well-being. A tendency towards increased general well-being was observed in both studies ABIHR-I and ABIHR-II, but again without significance. Further trials with a power-calculated sample size are needed.

Both groups did not differ significantly in terms of pain at rest (AB group, 17.1; No-AB group, 16.6; Table 2). However, three people in the No-AB group received a peridural catheter for the administration of pain medication. Only one patient in the AB group had one. As this catheter is usually removed within the first week after surgery (the time of removal was not documented), we assume that the primary endpoint with pain at rest on the 14th POD was not affected. On the other hand, a pain-reducing effect of AB within the first days after surgery may be masked by more frequent use of the peridural catheter in the No-AB group. Furthermore, following IHR, a pain-reducing effect of AB was found in the ABIHR-I trial and was reported by patients in the past. A meta-analysis from 2021 also found a pain-reducing effect of AB following laparotomies [3, 5, 18].

Our results suggest that wearing an AB after IHR may affect the SSI rate. Further studies are mandatory to confirm these findings because if the AB does not have to be prescribed it could lead to a reduction in costs. In 2019, a total of 48,793 people underwent surgery for incisional hernia [19]. The aforementioned survey showed that 70% of surgical departments recommend wearing an AB [2]. This bandage costs about 40 euros. In summary, the health care system in Germany is confronted with costs amounting to 1,366,000 euros annually.

As study limitations, the ABIHR-II studies, which began in 2019, faced recruitment challenges due to the COVID-19 pandemic. An amendment was requested in 2021 to reduce the pilot study's sample size from 60 to 50 patients due to the pandemic's impact on elective surgical programs in major hospitals in Germany. Due to an organizational error, one more patient than planned was enrolled in the study after the sample size was reduced (*n* = 51 instead of *n* = 50). For estimation of the sample size, we used the publication of Christoffersen et al. (2014) [20]. The authors enrolled 54 patients and examined the effect on abdominal binders following laparoscopic umbilical and epigastric hernia repair. We enrolled 51 patients, unfortunately our dropout rate was higher. Thus, from our perspective, we chose an appropriate sample size for a pilot study.

Unfortunately, the SSI was not further differentiated due to a lack of a 30-day follow-up and the instruction to do so (the protocol states: wound infection: yes or no). The study relied on subjective measures, such as the VAS for pain at rest and mobility. Objective measures, such as pain medication intake and the six-minute walk test, could improve the study's reliability and generalizability [21]. Future studies with power-calculated sample size, stratified randomization (hernia size) using the same mesh should consider these limitations to achieve more robust and reliable results.

## Conclusion

Wearing an AB did not have an impact on pain and seroma formation rate but it may reduce the rate of postoperative SSI within the first 14 days after surgery. Further trials are mandatory to confirm these findings.

### Supplementary Information

Below is the link to the electronic supplementary material.

Supplementary file1 Table S1 Univariate analysis on baseline characteristics and perioperative data (intention-to-treat population) (DOCX 23 KB)

Supplementary file2 Table S2 Summarized data on EHS incisional hernia classification of the per-protocol population (DOCX 14 KB)

Supplementary file3 Table S3 Detailed information on endpoint data of the per-protocol population (DOCX 22 KB)

Supplementary file4 Figure S1 VAS scoring of general well-being, limited mobility, pain at rest, and the seroma size on the 1st, 2nd, 3rd, 4th, and 14th POD of the intention-to-treat population is depicted. General well-being, limited mobility, and pain at rest were measured using a VAS (y-axis). The seroma size (y-axis, cm3) was documented using ultrasound imaging. The appearance of an SSI was documented as Yes or No. (PNG 54 KB)

## Data Availability

Data are available on request.
