# Supplementary material for: The effect of an abdominal binder on postoperative outcome after open incisional hernia repair in sublay technique: a multicenter, randomized pilot trial (ABIHR-II)
Source: Hernia. 2023 Jul 19;27(5):1263–71. doi: 10.1007/s10029-023-02838-4 (PMC10533646; doi:10.1007/s10029-023-02838-4)
Supplement: Supplementary file 1 — Supplementary file1 Table S1 Univariate analysis on baseline characteristics and perioperative data (intention-to-treat population) (DOCX 23 KB) [file 10029_2023_2838_MOESM1_ESM.docx]

**Table S1** Univariate analysis on baseline characteristics and perioperative data (Intention-to-treat population)

| **Variable** | **n = 51^1^** | **No-AB group, n = 26^1^** | **AB group, n = 25^1^** | **p-value^2^** | **t/X² (df)** | **Effect Size** |
| --- | --- | --- | --- | --- | --- | --- |
| Gender |  |  |  | >0.99 | 0.02 (1) | 0.020 |
| *female* | 25 (49.0%) | 13 (50.0%) | 12 (48.0%) |  |  |  |
| *male* | 26 (51.0%) | 13 (50.0%) | 13 (52.0%) |  |  |  |
|  |  |  |  |  |  |  |
| Age *years* | 61.4 (13.7) | 59.3 (14.2) | 63.6 (13.1) | 0.28 | -1.1 (49) | -0.309 |
|  |  |  |  |  |  |  |
|  |  |  |  |  |  |  |
| BMI *kg/m^2^* | 31.0 (5.0) | 31.2 (5.3) | 30.8 (4.7) | 0.81 | 0.25 (49) | 0.069 |
|  |  |  |  |  |  |  |
| ASA score |  |  |  | 0.20 | 3.21 (2) | 0.251 |
| I | 1 (2.0%) | 1 (3.9%) | 0 (0.0%) |  |  |  |
| II | 33 (64.7%) | 19 (73.1%) | 14 (56.0%) |  |  |  |
| III | 17 (33.3%) | 6 (23.1%) | 11 (44.0%) |  |  |  |
|  |  |  |  |  |  |  |
|  |  |  |  |  |  |  |
| Operating time  *minutes* | 137.8 (60.3) | 135.5 (56.5) | 140.0 (64.9) | 0.80 | -0.26 (47) | -0.074 |
| Missing data | 2 | 2 | 0 |  |  |  |
| Duration of hospital stay *days* | 8.7 (7.6) | 9.5 (9.7) | 7.9 (5.0) | 0.48 | 0.72 (46) | 0.207 |
| Missing | 3 | 3 | 0 |  |  |  |
|  |  |  |  |  |  |  |
| Surgeons experience years |  |  |  | 0.56 | 3.91 (5) | 0.285 |
| <5 | 1 (2.1%) | 0 (0.0%) | 1 (4.2%) |  |  |  |
| 5-10 | 11 (22.9%) | 5 (20.8%) | 6 (25.0%) |  |  |  |
| >10 | 11 (22.9%) | 7 (29.2%) | 4 (16.7%) |  |  |  |
| >20 | 20 (41.7%) | 10 (41.7%) | 10 (41.7%) |  |  |  |
| >30 | 1 (2.1%) | 1 (4.2%) | 0 (0.0%) |  |  |  |
| >40 | 4 (8.33%) | 1 (4.2%) | 3 (12.5%) |  |  |  |
| Missing | 3 | 2 | 1 |  |  |  |
|  |  |  |  |  |  |  |
| Mesh size  *cm^2^* | 566.9 (332.8) | 626,7 (341.1) | 507.1 (320.1) | 0.22 | 1.25 (46) | 0.362 |
|  | 3 | 2 | 1 |  |  |  |
| Component separation *yes* | 8 (17.0%) | 4 (16.7%) | 4 (17.4%) | >0.99 | 0.00 | 0.010 |
| Missing | 4 | 2 | 2 |  |  |  |
| Relapse if an incisional hernia |  |  |  | >0.99 | 0.00 (1) | 0.010 |
| No | 41 (80.4%) | 21 (80.8%) | 20 (80.0%) |  |  |  |
| Yes  Reoperation rate  Missing | 10 (19.6%)  5 (10.8%)  5 | 5 (19.2%)  2 (8.7%)  3 | 5 (20.0%)  3 (13.0%)  2 | >0.99 | 0.22 (1) |  |
|  |  |  |  |  |  | 0.421 |

^1^n (%); Mean value (Standard deviation)

^2^Chi-Quadrat-Test for independence; T-test

AB abdominal binder; ASA American Society of Anesthesiologists; BMI body mass index

Data are presented as mean (standard deviation) for continuous variables or total number (percentages) for categorical variables.

# Effect sizes are presented as Cohen’s d and Cramer’s V for independent t-tests and contingency tables, respectively.
