# Supplementary material for: The effect of an abdominal binder on postoperative outcome after open incisional hernia repair in sublay technique: a multicenter, randomized pilot trial (ABIHR-II)
Source: Hernia. 2023 Jul 19;27(5):1263–71. doi: 10.1007/s10029-023-02838-4 (PMC10533646; doi:10.1007/s10029-023-02838-4)
Supplement: Supplementary file 2 — Supplementary file2 Table S2 Summarized data on EHS incisional hernia classification of the per-protocol population (DOCX 14 KB) [file 10029_2023_2838_MOESM2_ESM.docx]

# Table S2 Summarized data on EHS incisional hernia classification of the per-protocol population

| **EHS classification** | n = 40^1^ | **No-AB group**, n = 19^1^ | **AB group**, n = 21^1^ |
| --- | --- | --- | --- |
| L1-2 W1 | 1,0 (2,5%) | 1,0 (5,3%) | 0,0 (0,0%) |
| L1 W3 | 1,0 (2,5%) | 0,0 (0,0%) | 1,0 (4,8%) |
| L2 W2 | 1,0 (2,5%) | 0,0 (0,0%) | 1,0 (4,8%) |
| M1-2 W2 | 1,0 (2,5%) | 0,0 (0,0%) | 1,0 (4,8%) |
| M1-2 W3 | 1,0 (2,5%) | 0,0 (0,0%) | 1,0 (4,8%) |
| M1-3 W1 | 1,0 (2,5%) | 0,0 (0,0%) | 1,0 (4,8%) |
| M1-3 W2 | 1,0 (2,5%) | 1,0 (5,3%) | 0,0 (0,0%) |
| M1-5 W2 | 1,0 (2,5%) | 1,0 (5,3%) | 0,0 (0,0%) |
| M1 W2 | 1,0 (2,5%) | 0,0 (0,0%) | 1,0 (4,8%) |
| M2-3 L2 W1 | 1,0 (2,5%) | 1,0 (5,3%) | 0,0 (0,0%) |
| M2-3 W1 | 2,0 (5,0%) | 1,0 (5,3%) | 1,0 (4,8%) |
| M2-3 W2 | 3,0 (7,5%) | 3,0 (15,8%) | 0,0 (0,0%) |
| M2-3 W3 | 3,0 (7,5%) | 3,0 (15,8%) | 0,0 (0,0%) |
| M2-4 L2 W3 | 1,0 (2,5%) | 0,0 (0,0%) | 1,0 (4,8%) |
| M2-4 W2 | 5,0 (12,5%) | 2,0 (10,5%) | 3,0 (14,3%) |
| M2-4 W3 | 4,0 (10,0%) | 1,0 (5,3%) | 3,0 (14,3%) |
| M2-5 W2 | 2,0 (5,0%) | 1,0 (5,3%) | 1,0 (4,8%) |
| M2-5 W3 | 1,0 (2,5%) | 1,0 (5,3%) | 0,0 (0,0%) |
| M2 W1 | 1,0 (2,5%) | 1,0 (5,3%) | 0,0 (0,0%) |
| M3 W1 | 2,0 (5,0%) | 0,0 (0,0%) | 2,0 (9,5%) |
| M3 W2 | 6,0 (15,0%) | 2,0 (10,5%) | 4,0 (19,0%) |
| ^1^n (%) | | | |
