# Supplementary material for: The effect of an abdominal binder on postoperative outcome after open incisional hernia repair in sublay technique: a multicenter, randomized pilot trial (ABIHR-II)
Source: Hernia. 2023 Jul 19;27(5):1263–71. doi: 10.1007/s10029-023-02838-4 (PMC10533646; doi:10.1007/s10029-023-02838-4)
Supplement: Supplementary file 3 — Supplementary file3 Table S3 Detailed information on endpoint data of the per-protocol population (DOCX 22 KB) [file 10029_2023_2838_MOESM3_ESM.docx]

**Table S3** Detailed information on endpoint data of the Per-protocol population

| Study arm | POD | Endpoints | N | median | mean | sd |  |
| --- | --- | --- | --- | --- | --- | --- | --- |
| No-AB group | POD 1 | Pain at rest | 18 | 35.0 | 35.6 | 24.2 |  |
| No-AB group | POD 2 | Pain at rest | 18 | 20.0 | 27.8 | 26.4 |  |
| No-AB group | POD 3 | Pain at rest | 16 | 23.0 | 29.6 | 20.2 |  |
| No-AB group | POD 4 | Pain at rest | 18 | 20.0 | 22.9 | 25.9 |  |
| No-AB group | POD 14 | Pain at rest | 19 | 3.0 | 17.1 | 24.7 |  |
| No-AB group | POD 1 | Limited mobility | 18 | 65.5 | 63.2 | 25.5 |  |
| No-AB group | POD 2 | Limited mobility | 18 | 45.0 | 43.6 | 24.8 |  |
| No-AB group | POD 3 | Limited mobility | 16 | 50.0 | 43.1 | 24.9 |  |
| No-AB group | POD 4 | Limited mobility | 18 | 30.0 | 33.2 | 24.8 |  |
| No-AB group | POD 14 | Limited mobility | 19 | 20.0 | 31.1 | 25.9 |  |
| No-AB group | POD 1 | General well-being | 18 | 57.5 | 56.2 | 25.8 |  |
| No-AB group | POD 2 | General well-being | 18 | 63.0 | 67.6 | 18.7 |  |
| No-AB group | POD 3 | General well-being | 16 | 73.0 | 70.7 | 19.1 |  |
| No-AB group | POD 4 | General well-being | 18 | 80.0 | 73.6 | 22.7 |  |
| No-AB group | POD 14 | General well-being | 19 | 80.0 | 72.4 | 26.0 |  |
| No-AB group | POD 1 | Seroma size (cm^3^) | 17 | 0.0 | 2.3 | 7.4 |  |
| No-AB group | POD 2 | Seroma size (cm^3^) | 18 | 0.0 | 0.6 | 1.7 |  |
| No-AB group | POD 3 | Seroma size (cm^3^) | 17 | 0.0 | 0.8 | 2.9 |  |
| No-AB group | POD 4 | Seroma size (cm^3^) | 18 | 0.0 | 0.0 | 0.1 |  |
| No-AB group | POD 14 | Seroma size (cm^3^) | 18 | 0.8 | 32.9 | 56.7 |  |
| No-AB group | POD 1 | SSI | 18 | 0.0 | 0.0 | 0.0 |  |
| No-AB group | POD 2 | SSI | 18 | 0.0 | 0.0 | 0.0 |  |
| No-AB group | POD 3 | SSI | 17 | 0.0 | 0.0 | 0.0 |  |
| No-AB group | POD 4 | SSI | 18 | 0.0 | 0.0 | 0.0 |  |
| No-AB group | POD 14 | SSI | 18 | 0.0 | 0.3 | 0.5 |  |
| AB group | POD 1 | Pain at rest | 21 | 40.0 | 40.2 | 21.9 |  |
| AB group | POD 2 | Pain at rest | 21 | 30.0 | 30.2 | 18.6 |  |
| AB group | POD 3 | Pain at rest | 20 | 20.0 | 23.1 | 16.9 |  |
| AB group | POD 4 | Pain at rest | 20 | 20.0 | 25.7 | 22.3 |  |
| AB group | POD 14 | Pain at rest | 21 | 10.0 | 16.6 | 17.7 |  |
| AB group | POD 1 | Limited mobility | 21 | 50.0 | 48.6 | 27.3 |  |
| AB group | POD 2 | Limited mobility | 21 | 34.0 | 37.1 | 21.2 |  |
| AB group | POD 3 | Limited mobility | 20 | 20.0 | 29.1 | 21.4 |  |
| AB group | POD 4 | Limited mobility | 20 | 20.0 | 23.8 | 19.9 |  |
| AB group | POD 14 | Limited mobility | 21 | 20.0 | 20.2 | 19.5 |  |
| AB group | POD 1 | General well-being | 21 | 60.0 | 62.5 | 20.0 |  |
| AB group | POD 2 | General well-being | 21 | 72.0 | 67.1 | 23.8 |  |
| AB group | POD 3 | General well-being | 20 | 80.0 | 74.5 | 14.0 |  |
| AB group | POD 4 | General well-being | 20 | 85.0 | 80.9 | 15.3 |  |
| AB group | POD 14 | General well-being | 21 | 90.0 | 84.8 | 18.5 |  |
| AB group | POD 1 | Seroma size (cm^3^) | 20 | 0.0 | 0.0 | 0.0 |  |
| AB group | POD 2 | Seroma size (cm^3^) | 21 | 0.0 | 0.8 | 3.5 |  |
| AB group | POD 3 | Seroma size (cm^3^) | 21 | 0.0 | 2.7 | 8.8 |  |
| AB group | POD 4 | Seroma size (cm^3^) | 21 | 0.0 | 40.5 | 156.5 |  |
| AB group | POD 14 | Seroma size (cm^3^) | 21 | 3.1 | 129.4 | 314.5 |  |
| AB group | POD 1 | SSI | 21 | 0.0 | 0.0 | 0.0 |  |
| AB group | POD 2 | SSI | 21 | 0.0 | 0.0 | 0.0 |  |
| AB group | POD 3 | SSI | 20 | 0.0 | 0.0 | 0.0 |  |
| AB group | POD 4 | SSI | 20 | 0.0 | 0.0 | 0.0 |  |
| AB group | POD 14 | SSI | 21 | 0.0 | 0.0 | 0.2 |  |

AB abdominal binder; POD postoperative Day; sd standard deviation; SSI surgical site infection

General well-being, pain at rest and limited mobility were measured using the Visual Analog Scale.

The appearance of a wound infection was clinical documented as *Yes* or *No*
