# Supplementary figures and images for: The effect of an abdominal binder on postoperative outcome after open incisional hernia repair in sublay technique: a multicenter, randomized pilot trial (ABIHR-II)
Source: Hernia. 2023 Jul 19;27(5):1263–71. doi: 10.1007/s10029-023-02838-4 (PMC10533646; doi:10.1007/s10029-023-02838-4)

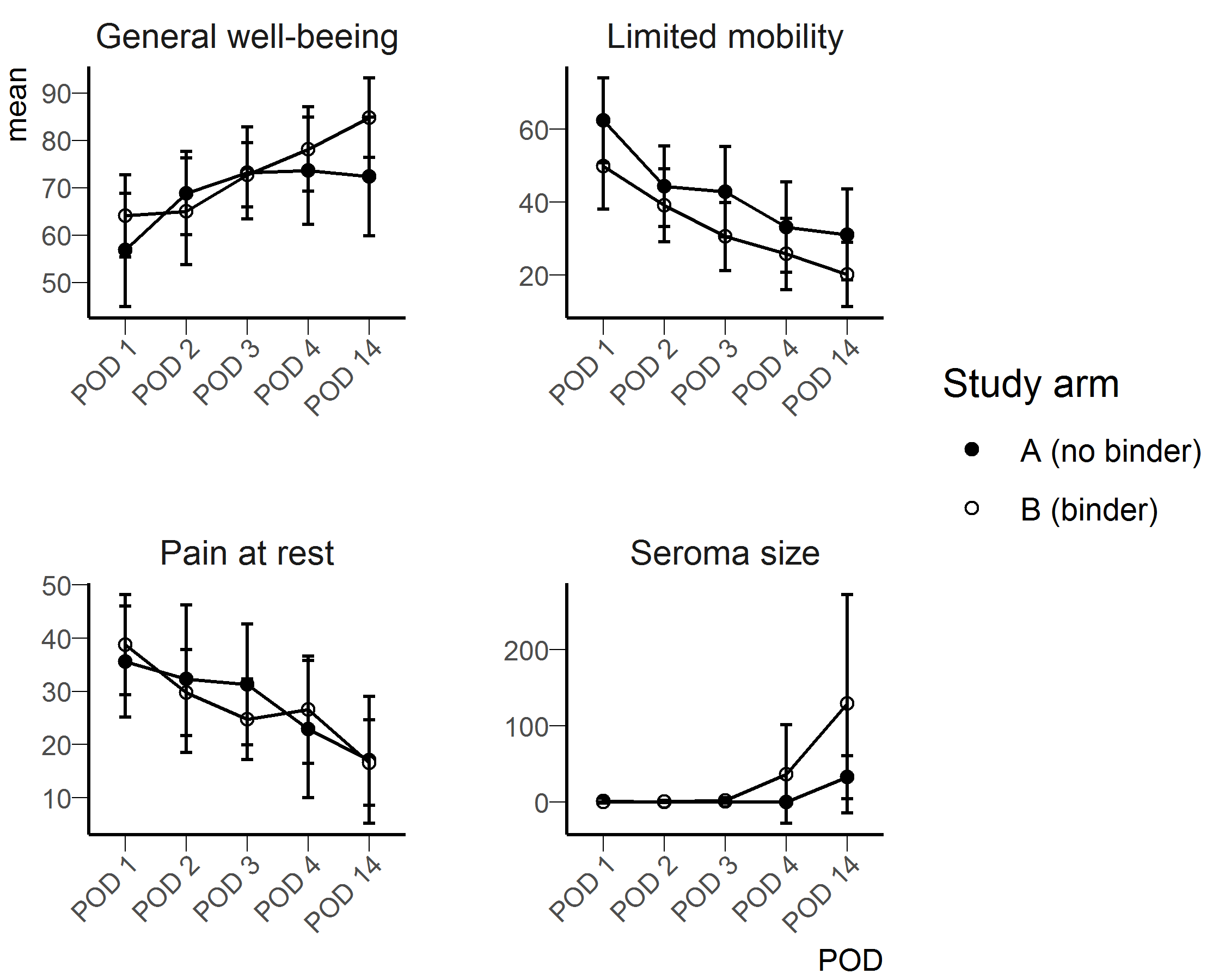

Supplement: Supplementary file 4 — Supplementary file4 Figure S1 VAS scoring of general well-being, limited mobility, pain at rest, and the seroma size on the 1st, 2nd, 3rd, 4th, and 14th POD of the intention-to-treat population is depicted. General well-being, limited mobility, and pain at rest were measured using a VAS (y-axis). The seroma size (y-axis, cm3) was documented using ultrasound imaging. The appearance of an SSI was documented as Yes or No. (PNG 54 KB) [file 10029_2023_2838_MOESM4_ESM.png]
